# Novel *ECHS1* mutations in Leigh syndrome identified by whole-exome sequencing in five Chinese families: case report

**DOI:** 10.1186/s12881-020-01083-1

**Published:** 2020-07-16

**Authors:** Dan Sun, Zhimei Liu, Yongchu Liu, Miaojuan Wu, Fang Fang, Xianbo Deng, Zhisheng Liu, Liang Song, Kei Murayama, Chunhua Zhang, Yuanyuan Zhu

**Affiliations:** 1 Department of Pediatric Neurology, Wuhan Children’s Hospital, Tongji Medical College, Huazhong University of Science & and Technology, Wuhan, 430016 China; 2 Department of Neurology, Beijing Children’s Hospital, Capital Medical University, National Center for Children’s Health, Beijing, 100045 China; 3 Aegicare (Shenzhen) Technology Co., Ltd., Shenzhen, 518110 China; 4 School of Medicine, Jianghan University, Wuhan, 430056 China; 5 Radiology Department, Union Hospital, Huazhong University of Science and Technology, Wuhan, 430056 China; 6 Third People’s Hospital of Hubei Province, Wuhan, 430030 China; 7 Center for Medical Genetics Department of Metabolism, Chiba Children’s Hospital, Chiba, 2660007 Japan; 8 MILS International, Yokohama, 2220033 Japan

**Keywords:** Leigh syndrome, *ECHS1*, Whole-exome sequencing, Case report

## Abstract

**Background:**

Short-chain enoyl-CoA hydratase deficiency (ECHS1D), also known as ECHS1 deficiency, is a rare inborn metabolic disorder with clinical presentations characterized by Leigh syndrome (LS). Thirty-four different pathogenic mutations have been identified from over 40 patients to date.

**Case presentation:**

Here, we report five Chinese patients with clinical syndromes typified as LS. Despite different initial symptoms, all patients presented developmental regression, dystonia, common radiological features such as symmetrical bilateral brain abnormalities, and similar metabolic results such as elevated plasma lactate and 2,3-dihydroxy-2-methylbutyrate. Utilizing whole-exome sequencing (WES), we identified eight distinct variants in *ECHS1*, with six novel variants, and the remaining two variants have been previously reported. Interestingly, one of the six novel variants, c.463G > A (p.Gly155Ser), was detected in three patients from unrelated families, suggesting a potential founder effect already described for a few mutations in LS. Incorporating both genetic analysis and medical results, including magnetic resonance imaging (MRI), electroencephalography (EEG), and biochemical testing, our study enriched the mutation spectrum of the *ECHS1* gene and confirmed the phenotypic presentations of LS.

**Conclusions:**

The severity of ECHS1 deficiency seems to vary. It was affected by both genetics and external environmental factors that lead to increased metabolism. Our study enriched the mutation spectrum of the *ECHS1* gene, confirmed the phenotypic presentations, and highlighted the importance of the valine catabolic pathway in Leigh syndrome. Further studies are required to examine the potential founder mutation c.463G > A (p.Gly155Ser) and the role of ECHS1 in relevant pathways.

## Background

Mitochondrial short-chain enoyl-CoA hydratase-1 deficiency, also known as ECHS1 deficiency, is an autosomal recessive inborn metabolic disorder commonly presenting after birth or early in life. It is typically characterized by developmental delay, regression, high levels of lactic acid, and abnormalities in the basal ganglia of the brain [1]. These clinical symptoms are consistent with Leigh syndrome (LS) or Leigh-like syndrome, which are rare heterogeneous progressive neurodegenerative disorders. In particular, T_2_ bilateral hyperintensities are characteristic of cranial magnetic resonance imaging (MRI) in LS [2, 3].

*ECHS1* gene mutations were first discovered in two infant siblings in 2014 and reported to be one of the causes of LS [1]. These mutations are involved in the metabolic pathway of essential amino acids such as valine, as well as affecting the second step of mitochondrial fatty acid beta-oxidation (FAO). The reported clinical and biochemical characteristics of ECHS1 deficiency strongly indicated that the common pathological mechanism of ECHS1 deficiency is dysfunction of the valine catabolic pathway. The valine catabolic pathway has been elucidated to involve five enzymes. ECHS1 is responsible for the fourth step of valine degradation, which is to convert unsaturated trans-2-enoyl-CoA species, such as methacrylyl-CoA and acryloyl-CoA, to the corresponding 3(S)-hydroxyacyl-CoA [4]. The deficiency of ECHS1 leads to the accumulation of methacrylyl-CoA and acryloyl-CoA, two toxic intermediates that are highly reactive with sulfhydryl groups that are suspected to cause brain pathology and the relevant biochemical patterns [5].

Recently, Sharpe and McKenzie performed a comprehensive review of all reported cases of ECHS1 deficiency [2]. Briefly, thirty-four different mutations from 42 patients have been described to date. Most identified mutations are missense, and the majority of reported cases are compound heterozygous. Apart from c.476A > G (p.Gln159Arg), which has been found in multiple unrelated ECHS1-deficient patients of diverse ethnic origins [4, 6–9], no other hotspot mutations have been characterized.

Here, we report five patients from different Chinese families. They were diagnosed with LS with typical clinical symptoms such as developmental regression, dystonia, symmetrical bilateral abnormalities, and biochemical features such as increased 2,3-dihydroxy-2-methylbutyrate and elevated plasma lactate, which strongly indicated ECHS1 deficiency. Whole-exome sequencing (WES) was performed on five patients to precisely identify the disease-causing genes and variants. Eight mutations in the *ECHS1* gene were detected, six of which are novel. In particular, one of the novel mutations, c.463G > A (p.Gly155Ser), was detected in three of the five patients from unrelated families. Considering that the studied patients were all from the same region of China, it is a strong indication of a founder effect that has been reported in a few variants of ECHS1 deficiency in previous studies [4, 7, 9]. Our study enriched the mutation spectrum of the *ECHS1* gene, confirmed the phenotypic presentations, and highlighted the importance of the valine catabolic pathway in Leigh syndrome.

## Case presentation

### Subjects

Five patients from different families were diagnosed with LS in the Department of Pediatrics of Tongji Hospital in Wuhan and the Department of Neurology, Beijing Children’s Hospital, Capital Medical University between 2016 and 2018. The study protocols were approved by ethics committees of both hospitals. Informed consent for routine and investigative genetic studies was obtained from parents of all studied patients and families.

### Metabolite analysis

According to previous studies, urine 2,3-dihydroxy-2-methylbutyrate, a special indicator on routine organic acid analysis, was measured for each studied patient [4, 10]. Acylcarnitine analysis of both butyl derivatives and plasma lactate/alanine/proline were also performed.

### Mitochondrial respiratory chain (MRC) enzyme activity measurement

Two patients (patients 2 and 3) were arranged to measure enzyme activity by cultured skin fibroblasts. The mitochondrial marker enzyme citrate synthase (CS) and MRC complexes I, II, II + III, III, IV activity were detected in isolated mitochondria from skin fibroblasts, as described previously [11]. Enzyme activities of MRC complexes were calculated by the mean percentage of normal control relative to appropriate reference enzyme activities (such as CS). Enzyme activities in the cell line were defined as being decreased at < 40% [12].

### Oxygen consumption rate (OCR) measurement

The OCR of fibroblasts was measured in two patients (patients 2 and 3) by an XF96 Extracellular Flux Analyzer (Seahorse Bioscience, Billerica, MA, USA). Samples were prepared according to descriptions in previous studies [13, 14]. Patient samples were measured together with two controls in each run. Patient fibroblast cell lines and controls were seeded in at least 14 wells of two XF96 cell culture microplates (Seahorse Bioscience) at a cell density of 20,000 per 80 μL of growth medium in each well and incubated overnight (5% CO_2_, 37 °C). The growth medium was then replaced with 160 μl of 25 mM glucose medium or 10 mM galactose medium the following day. The microplate was placed into a CO_2_-free incubator at 37 °C for 60 min before measurement. After the measurement of basal OCR, 10 μM oligomycin, 4 μM carbonyl cyanide phenylhydrazone (FCCP), and 20 μM rotenone were added, followed by OCR recording upon each addition [13]. The maximum respiration rate (MRR) corresponds to the OCR after FCCP injection minus rotenone-insensitive OCR. MRR was denoted as a percentage relative to the average of the controls. A reduction of less than 71.6% was considered a significant decline [14].

### Genetic analysis

Due to the genetic heterogeneity where LS can be caused by genetic abnormalities from more than 75 genes, WES and analysis for the detection of variants were performed on all five patients. Briefly, genomic DNA was extracted from whole blood samples. Whole exons and flanking intronic sequences were captured by an Agilent Sure-Select Human All Exon Kit v6, followed by high-throughput sequencing on the Illumina HiSeq 2000 platform with 150-bp paired-end reads. Bioinformatic analysis was carried out with public software and a self-developed pipeline. Specifically, all cleaned data after trimming were aligned against the human reference genome build hg19 using BWA [15]. The average depths of WES data ranged from 95x to 171x across five patients.

Single nucleotide variants (SNVs) and indels were then discovered by HaplotypeCaller of GATK, followed by variant annotation through ANNOVAR [16], integrating customized databases such as ClinPred [17]. The analysis was restricted to coding and flanking intronic regions (±20 bp) where good data coverage is normally guaranteed. Variants with minor allele frequency (MAF) greater than 1% in any of the gnomAD, ExAC, 1000 Genomes Project, and ESP6500 databases were excluded from subsequent analysis. Filtering and prioritizing were then performed to look into potential detrimental variants such as nonsense, missense, frameshift, and variants impacting splice for variant interpretation and classification according to American College of Medical Genetics (ACMG) guidelines [18].

To confirm the mutations identified by WES, fragments covering the mutation site in *ECHS1* were amplified by PCR followed by Sanger sequencing. PCR was conducted with Premix Taq™ Hot Start Version/TaKaRa LA Taq® with GC Buffer (Takara, Osaka, Japan) following the following protocol: 95 °C for 2 min, then 35 cycles consisting of 95 °C for 30 s, 60 °C for 30 s and 72 °C for 30 s, 72 °C for 5 min in the end. PCR products were purified and sequenced on the ABI 3730 DNA Analyzer using the BigDye™ Terminator Cycle Sequencing Kit (Applied Biosystems, Foster, CA, USA).

## Results

### Clinical features, brain MRI examinations, and biochemical investigations

The studied patients were from five different and unrelated Chinese families with healthy and nonconsanguineous parents. Their ages of onset ranged from birth to 21 months, with diverse initial symptoms. However, they presented similar clinical features, MRI findings, and biochemical results, such as developmental regression; bilateral abnormal signals, especially around basal ganglia; and elevated serum lactate, respectively. These characteristics strongly indicated that they have the same disease or syndrome. Table 1 summarizes the general information, clinical features, brain MRI findings, and biochemical examination results of all patients. We describe each patient in detail in the following section.

**Table 1:** Summary of general information, clinical features, brain magnetic resonance imaging and biochemical examinations of five patients

	Patient 1	Patient 2	Patient 3	Patient 4	Patient 5
**General information and clinical features**					
Gender	Male	Female	Male	Male	Male
Age of onset	6 months	21 months	17 months	At birth	10 months
Current age	4 years and 7 months	6 years	29 months	8 months	19 months
Initial presentation	Nystagmus	regression	Episodes of dystonia	Paroxysmal dyskinesia	Diarrhea, eyes on the turn, myotonia of the lower limb
Central Nervous System	Global developmental delay (HP:0001263); Developmental regression (HP:0002376); Nystagmus (HP:0000639); Extrapyramidal dyskinesia (HP:0007308); Babinski sign (HP:0003487); Dystonia (HP:0001332)	Developmental regression (HP:0002376); Dysarthria (HP:0001260); Dystonia (HP:0001332)	Dystonia (HP:0001332)	Motor deterioration (HP:0002333)	Developmental regression (HP:0002376)
**Brain magnetic resonance imaging**					
Globus pallidus	+	+	+	+	+
Putamen	+	+	–	+	+
Caudate nucleus	+	+	–	+	+
Brain stem	+	–	–	+	–
Summary	Symmetric lesions of the basal ganglia (HP:0007039); Brain atrophy (HP:0012444)	Symmetric lesions of the basal ganglia (HP:0007039)	Symmetric lesions of the basal ganglia (HP:0007039)	Symmetric lesions of the basal ganglia (HP:0007039)	Symmetric lesions of the basal ganglia (HP:0007039)
**Biochemical examination**					
Plasma lactate (mmol/L)	1.6- > 3.4 (0.5–2.2)	1.68 (0.5–2.2)	3.17- > 6.46 (0.5–2.2)	11.41 (0.5–2.2)	2.51 (0.5–2.2)
Plasma pyruvate (umol/L)	197–255	Not performed	Not performed	Not performed	Not performed
Acylcarnitine analysis (DBS)	Normal profile	Normal profile	Normal profile	Slight increase in C4OH	Normal profile
Organic acid analysis	Increases in 2,3-dihydroxy-2- Methylbutyrate (0.0446)	Increases in 3-hydroxyisovaleric acid and 2,3-dihydroxy-2- Methylbutyrate (0.0445)	Increases in 2,3-dihydroxy-2- Methylbutyrate	Increases in 2,3-dihydroxy-2- Methylbutyrate	Increases in pyruvate
Skin biopsy	Not performed	OCR:79%/78% (Glucose/Galactose medium) MRC enzyme activity measurement: no significance	OCR:56%/71% (Glucose/Galactose medium) MRC enzyme activity measurement: no significance	Not performed	Not performed

Patient 1 is currently 4 years and 7 months old. He was born by caesarean section at full term and exhibited normal developmental milestones until initial presentations of nystagmus at 6 months. Arrested development of being unable to sit steadily started to be noticed at the age of 8 months. He underwent the first brain MRI examination at 8 months and an enlarged subarachnoid space and hyperintensities of the right caudate head and bilateral globus pallidus were found in T2-weighted images. He then received rehabilitation training but without visible improvement. Global regression with gradually progressive extrapyramidal dyskinesia and dystonia was noted at the age of 10 months. His second brain MRI was carried out at 12 months because other symptoms started to appear. Bilateral lesions of the cerebral peduncle and basal ganglia, including the globus pallidus, caudate nucleus, and putamen, as well as brain atrophy, were observed.

His initial biochemical investigations showed marginally elevated lactate and pyruvate in plasma. The lactate/pyruvate ratio was measured, and the value was 13.3. However, no significant abnormality was found in the acylcarnitine analysis. Urine organic acid analysis also showed only a slightly abnormal increase in 2,3-dihydroxy-2-methylbutyrate.

Patient 2 was the only female child among the five patients. She is the first child of a family with a healthy half-sister from the same father. Her first manifestation was a sudden convulsion that lasted for 90 min at the age of 21 months. Development milestones were reported by parents to be normal previously. Blood gas analysis was carried out to show metabolic acidosis. She had her first brain MRI at admission and showed bilateral basal ganglia lesions that mainly involved the globus pallidus, where the right side was more severe than the left. No epileptic discharge was found in the EEG results. Global developmental regressions, such as motor and language, were manifested thereafter. She then received rehabilitation training, with visible but limited benefits, showing that she could walk unsteadily at 3 years old, as well as be capable of learning and understanding simple instructions but without active communication. She underwent brain MRI reexamination at the age of 4 years and 7 months. Bilateral lesions of the globus pallidus, caudate nucleus, and putamen were evident; however, the lesions of the globus pallidus were smaller than the previous examination.

The biochemical analysis showed that her plasma lactate was normal. For urine organic acid testing, 3-hydroxyisovaleric acid and 2,3-dihydroxy-2-methylbutyrate were abnormally increased. Acylcarnitine was at a normal level. Skin biopsy demonstrated normal OCR (79%/78%) and enzyme activity of the respiratory chain.

Patient 3 is also the first child in his family, with almost normal development milestones. At the age of 17 months, he started to manifest dystonia with an unexplained cause. The dystonia normally lasted for one minute but could be up to 30 min in severe cases. He appeared normal in between two dystonia phases. In addition, salivation and choking were ordinarily observed during water drinking. His biomedical tests showed metabolic acidosis and elevated serum lactate but no specific changes in organic acid and amino acid profiles. He also underwent two brain MRI examinations at the ages of 17 months and 21 months, presenting similar results with bilateral globus pallidus involvement.

His plasma lactate showed mild elevation. The level of 2,3-dihydroxy-2-methylbutyrate was increased; however, acylcarnitine was normal. Skin biopsy showed that OCR was abnormally decreased (56%/71%) but with essentially normal enzyme activity of the respiratory chain.

**Fig. 1:**
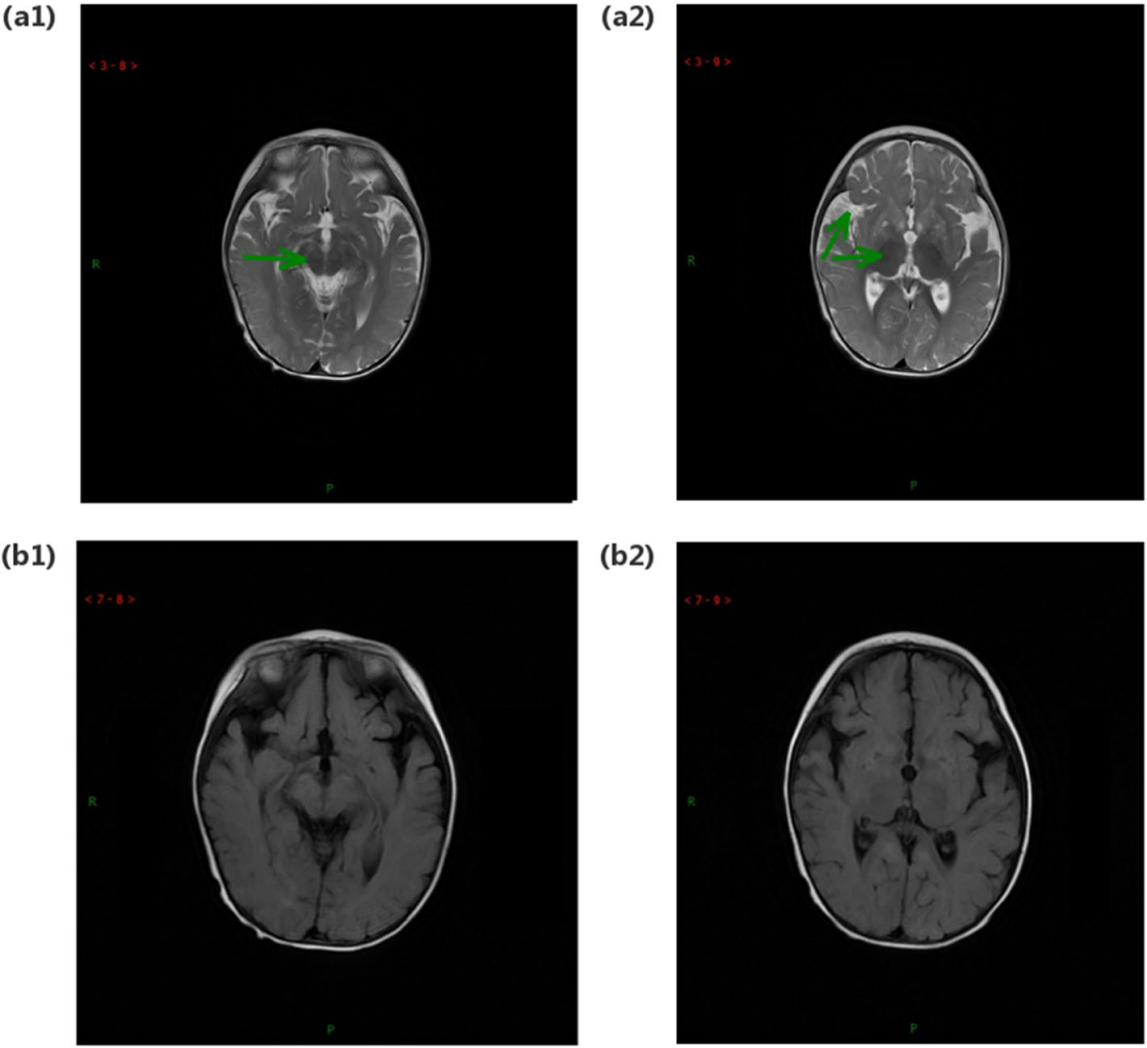
MRI and MRS of patient P4. Images (a1) and (a2) are T2-weighted images, with green arrows showing abnormal areas. (b1) and (b2) are T2-FLAIR images

Patient 4 was admitted to the hospital at 8 months old, the youngest child of the five patients. He is the second child in his family with a healthy brother and was delivered vaginally with paroxysmal dyskinesia at his birth. He was unable to raise his head at the moment of admission. He underwent a brain MRI examination twice. The results of the first study showed abnormal symmetrical bilateral signals in the basal ganglia, thalamus, and midbrain cerebral peduncle, as well as an enlarged subarachnoid space (see Fig. 1). The second examination was performed 17 days later and demonstrated that both brain stem and basal ganglia present abnormal bilateral signals.

In the biochemical analysis, plasma lactate and hydroxybutyrylcarnitine (C_4_OH) were elevated. In addition, 2,3-dihydroxy-2-methylbutyrate was increased based on the urine organic acid analysis. When he returned for review, at the age of 13 months, he was still unable to raise his head, sit steadily or speak. The level of serum lactate increased slightly.

**Fig. 2:**
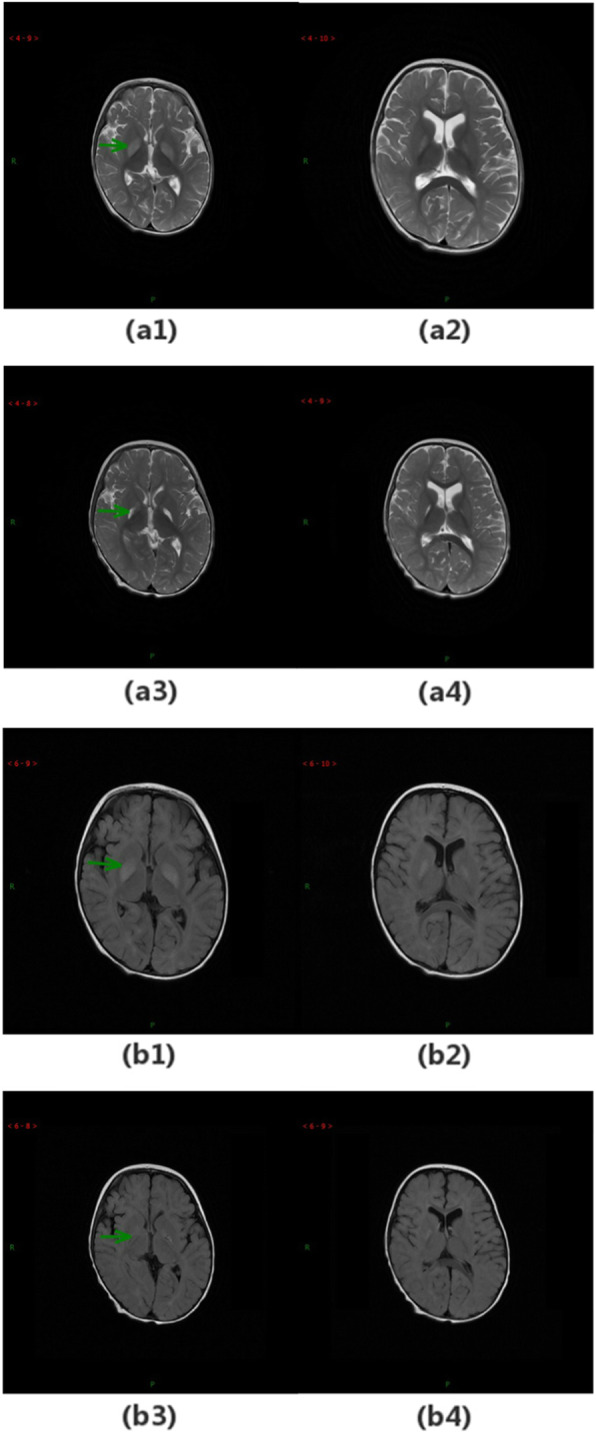
MRI images of patient P5 with green arrows showing abnormal areas. Images (a1–4) and (b1–4) are T2-weighted and T2-FLAIR images, respectively. Images (a1–2) and (b1–2) are the results of the first MRI, which show symmetric abnormal signals of the bilateral basal ganglia. Images (a3–4) and (b3–4) show the results of the second MRI, which were similar to the first MRI

Patient 5 was the younger of identical twins. He was admitted to our hospital at the age of 10 months with diarrhoea and myotonia of the lower limb, as well as developmental regression including intellectual, motor, speech, and language. A slightly elevated slow wave was found in the EEG. Brain MRI examination showed an asymmetric abnormal signal of the bilateral basal ganglia (see Fig. 2). Urine organic acid analysis revealed increased pyruvate. He still had difficulty controlling his head and body after 12 days of treatment. He then started rehabilitation training at the age of 15 months. During this period, he underwent the second MRI and EEG examinations, which still showed marked bilateral partial basal ganglia and massive profound fast waves, respectively. At 16 months, he was admitted to our hospital again with vomiting. His physical examination reported that he had appendicular motor hypertonia, mild genu valgum, and calcaneovalgus deformity, as well as being incapable of sitting steadily. The biochemical test showed an increased level of pyruvate in urine organic acid analysis and a slight elevation in plasma lactate levels but normal acylcarnitine levels. His twin brother presented syndromes similar to him but mildly.

### Genetic analysis

#### Family 1

**Fig. 3:**
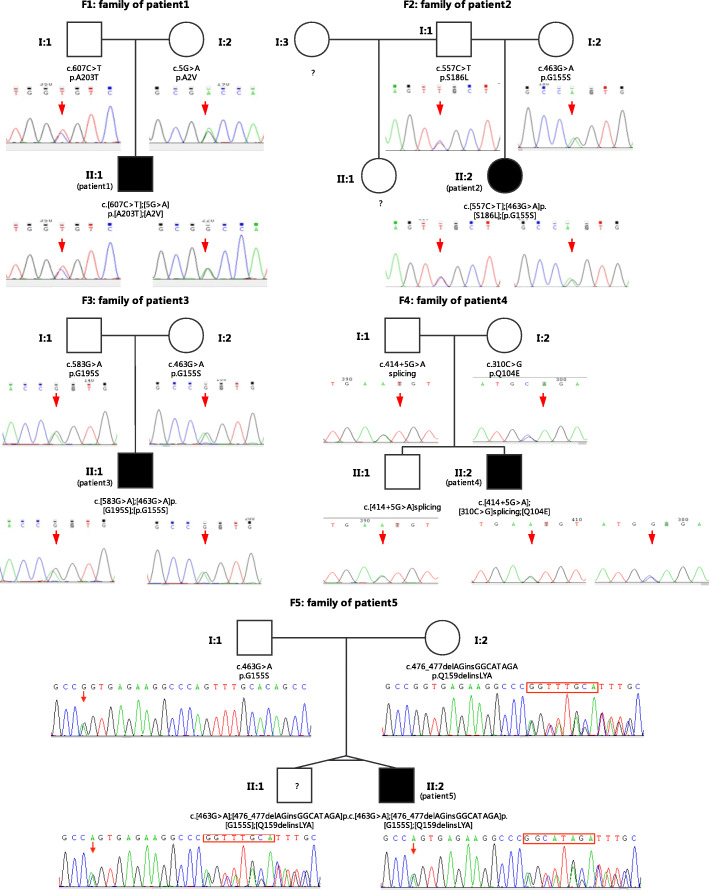
Genograms with Sanger confirmation results for the five studied families

One of the identified biallelic mutations of patient 1, c.5C > T (p.Ala2Val), in exon 1 of the *ECHS1* gene (NM_004092.3) has been previously reported to be likely pathogenic, with functional verification [19, 20]. The other mutation, c.607C > T (p.Ala203Thr), which is located in exon 5, is novel. Multiple lines of computer programs predict that the mutation is damaging. The population allele frequencies of both mutations are either much lower than 1% in or not even present in the population frequency databases (Table 2). We searched our in-house database, which consists of approximately 5000 samples, and found no samples with either mutation. Sanger sequencing was performed on the patients and their parents and confirmed that c.5G > A (p.Ala2Val) and c.607C > T (p.Ala203Thr) are inherited from his heterozygous mother and father, respectively (see Fig. 3).

**Table 2:** Results of *ECHS1* genetic analysis of the 5 patients

Patient	Variant ^a^	Origination ^b^	Exon/ Intron	Position (hg19)	Novel/ reported (PMID)	Population allele frequency ^c^			Pathogenicity Scores						
						gnomAD	ExAC	1000 Genomes	SIFT	Polyphen2_HDIV	Polyphen2_HVAR	LRT	dbscSNV	GERP (NR)	GERP (RS)
1	c.5C > T (p.A2V)	M	Exon 1	10:135186833	Reported (25393721)	1.02•10^−5^	0	–	0.005	0.011	0.011	–	–	3.75	2.8199
	c.607C > T (p.A203T)	P	Exon 5	10:135180405	novel	2.01•10^−5^	8.35•10^−6^	–	0	1	0.997	0	–	5.92	5.01
2	c.463G > A (p.G155S)	M	Exon 4	10:135182478	novel	1.99•10^−5^	13.32•10^−5^	2.0•10^− 4^	0.24	0.981	0.81	0	–	5.8099	5.8099
	c.557C > T (p.S186L)	P	Exon 5	10:135180455	novel	1.44•10^−4^	2.0•10^− 4^	2.0•10^− 4^	0.001	1	0.999	0	–	5.92	5.92
3	c.463G > A (p.G155S)	M	Exon 4	10:135182478	novel	1.99•10^−5^	13.32•10^−5^	2.0•10^−4^	0.24	0.981	0.81	0	–	5.8099	5.8099
	c.583G > A (p.G195S)	P	Exon 5	10:135180429	Reported (26000322)	1.02•10^−5^	8.35•10^−6^	–	0.009	1	1	0	–	5.92	5.92
4	c.310C > G (p.Q104E)	M	Exon 3	10:135183512	novel	–	–	–	0.201	0.012	0.027	0	–	5.63	3.7
	c.414 + 5G > A (splicing)	P	Intron 3	10:135183403	novel	–	–	–	–	–	–	–	0.9999	5.9	5.9
5	c.463G > A (p.G155S)	M	Exon 4	10:135182478	novel	1.99•10^−5^	13.32•10^−5^	2.0•10^−4^	0.24	0.981	0.81	0		5.8099	5.8099
	c.476_477delAGinsGGCATAGA (p.Q159delinsLYA)	P	Exon 4	10: 135182464	novel	–	–	–	–	–	–	–		5.8099	4.3849

^a^The transcript used is NM_004092.3

^b^“P” is short for Paternal, “M” is short for Maternal

^c^The population frequencies are the global frequencies (ALL) of the gnomAD, ExAC and 1000 Genomes databases. The dash ‘-‘denotes no records for the variant in the database

#### Family 2

Biallelic mutations, c.463G > A (p.Gly155Ser) and c.557C > T (p.Ser186Leu), were identified in patient 2. Neither has been previously reported. Similar to patient 1, the population allele frequencies of both mutations were much lower than 1% in all databases (Table 2). Multiple computer programs predicted that the two mutations are damaging. Sanger sequencing confirmed that her parents are both heterozygous for the *ECHS1* gene carrying variants c.463G > A (p.Gly155Ser) and c.557C > T (p.Ser186Leu), respectively.

#### Family 3

For patient 3, the identified variants were c.583G > A (p.Gly195Ser) and c.463G > A (p.Gly155Ser), which were in exon 5 and exon 4 of *ECHS1,* respectively. The first has been previously reported [7], with a population allele frequency lower than 1% for the global population in gnomAD. The second is the same as one of the identified mutations of patient 2. Multiple computer programs predicted that the two mutations are damaging. The patient’s father was confirmed to be heterozygous for c.583G > A in *ECHS1*, and his mother was confirmed to be heterozygous for c.463G > A.

#### Family 4

Unlike the mutations identified for the previous three patients, the intron mutation c.414 + 5G > A in intron 3 of the *ECHS1* gene was identified in patient 4. The computer programs (GERP, dbscSNV) predict that the site is conserved and that the mutation most likely leads to splicing alteration. The other variant identified is c.310C > G (p.Gln104Glu) in exon 3. Both are novel mutations that have never been reported in the literature or recorded in databases. Sanger sequencing confirmed that the variants were inherited from his father (c.414 + 5G > A) and mother (c.310C > G). Both parents are heterozygous for the *ECHS1* gene.

#### Family 5

An indel variant in exon 4 of *ECHS1*, c.476_477delAGinsGGCATAGA, which was inherited from his father, was identified. It has never been mentioned in previous studies, interpretations, or any databases. However, it is worth noting that c.476A > G (p.Gln159Arg) is a hotspot mutation for ECHS1D in ClinVar and was categorized as “pathogenic/likely pathogenic”. Taking both facts together into consideration, this variant can be regarded as a combination of two isolated mutations of c.476A > G (p.Gln159Arg) and c.478insCATAGA. Hence, it is rational to consider that this allele has a likely pathogenic variant. The other variant confirmed to originate from the patient’s mother is c.463G > A (p.Gly155Ser), which was also present in patients 2 and 3.

In summary, the five patients in our study were all compound heterozygous, with different variants in *ECHS1* alleles inherited from their parents. Sanger sequencing was carried out for each family to verify the variant inheritance (Fig. 3). The results of the genetic analysis are summarized in Table 2.

## Discussion and conclusions

In this study, we reported five patients presenting diverse initial symptoms but consistent later clinical features with LS. Regarding genetic heterogeneity, whole-exome sequencing was performed to uncover disease-causing genes and mutations.

During the genetic analysis, none of the five patients were found to have mutations in any previously reported nuclear genes associated with LS except for *ECHS1*. Mitochondrial DNA sequencing was also performed for patients 2 and 3 without any explainable mutations identified. Finally, we identified eight distinct mutations; six were novel, and the other two have been previously reported [7, 19]. As shown in Table 2, all detected mutations are extremely rare in public population databases, with a few of them not even present. In addition, none of these mutations were found in our in-house database with approximately 5000 samples. Each of these mutations is classified as pathogenic or detrimental by at least one or more prediction methods.

**Fig. 4:**
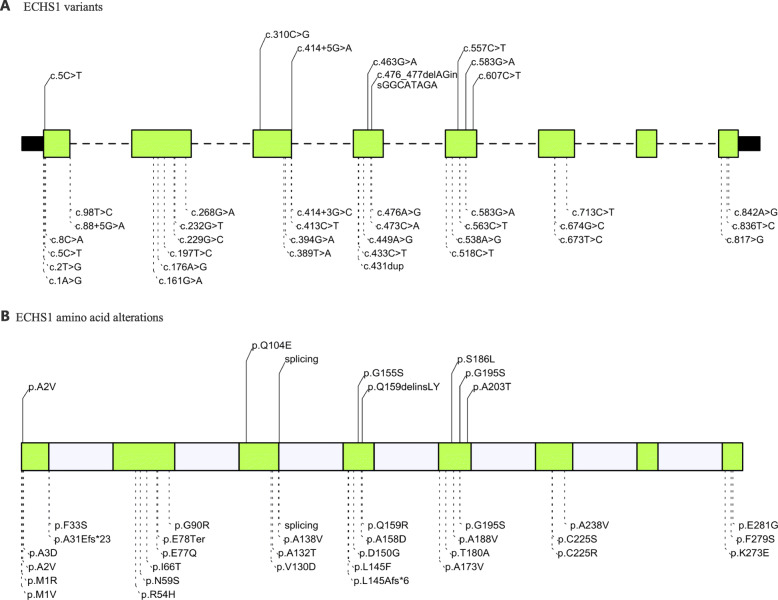
Summary of the variants and amino acid alterations of *ECHS1* gene related to Leigh syndrome. (A) The previously reported variants (below the bar with dashed lines) and the newly identified variants (above the bar with solid lines); (B) The previously reported amino acid alterations (below the bar with dashed lines) and the newly identified amino acid alterations (above the bar with solid lines)

Sharpe and McKenzie performed a comprehensive review of ECHS1D and collected all reported *ECHS1* mutations up to then in 2018 [2]. Carlston and colleagues updated the reported mutations in their case report of ECHS1D [21]. To date, 34 pathogenic *ECHS1* mutations have been identified, with 29 missense, 2 splicing, 2 frameshift and 1 nonsense [2, 21]. We summarized the currently reported mutation spectrum of the *ECHS1* gene in Fig. 4. The same mutational pattern was also observed in our study, where the major mutation type is missense. Specifically, six of the eight identified mutations in our study are missense, with one splicing and one indel. It is worth noting that one of the novel mutations, c.463G > A (p.Gly155Ser), was detected in three of the five studied patients. ClinVar interpretation of pathogenicity is “uncertain significance”. However, combining multiple supporting lines of evidence, such as extremely low population allelic frequency, located in a functional domain without benign variations, multiple computational supporting evidence of being deleterious, as well as being a missense mutation, which is the common mechanism of ECHS1D, together with the fact that it was detected in three unrelated families in our study, we strongly suggested that it be recategorized as pathogenic or likely pathogenic. Moreover, this is also an indication of a founder effect that requires in-depth research and has been mentioned in a few ECHS1D studies [4, 7].

Clinically, despite different initial presentations, all studied patients presented similar clinical syndromes, such as development regression, paroxysmal exercise-induced dystonia, and common radiological features, such as symmetrical bilateral abnormalities. Moreover, the plasma lactate of most patients was elevated, together with a normal acylcarnitine profile and elevated erythro-2,3-dihydroxy-2-methylbutyrate levels. All these symptoms are consistent with other studies reporting Leigh syndrome [2, 4, 22, 23].

ECHS1 is a mitochondrial matrix enzyme that catalyses multiple metabolic pathways, such as fatty acids and valine oxidation. Recent studies reported that *ECHS1* mutations are the causes of severe early-onset Leigh-like mitochondrial encephalopathy, accompanied by deafness, epileptic seizures, and optic atrophy, as well as feeding problems and cardiomyopathy [1, 2, 4]. To date, we still do not know this mechanism. According to the consistently elevated lactate level, it is speculated that the accumulation of harmful intermediate metabolites may lead to brain toxicity and disorders of mitochondrial energy metabolism [1, 2]. In some cases, the marked presence of metabolite 2-methyl-2,3-hydroxybutyric acid in urine organic acids has been detected. The metabolic pathway of 2-methyl-2,3-dihydroxybutyrate is currently unclear and needs more in-depth research in the field.

The severity of ECHS1 deficiency seems to vary. It is affected by both genetics and external environmental factors that lead to increased metabolism [7]. Among our patients, three showed a sharp increase in the severity of symptoms after acute viral infections. This may be due to the elevated energy consumption in the decomposition states. Compared with early reported lethal cases [24], the results of biochemical testing can be complex and diverse, and metabolic abnormalities are often nonspecific.

Elevated pyruvate and lactate accompanied by normal pyruvate-lactate ratios have been observed in a few patients with early-onset lactic acidosis. Due to the heterogeneity of the natural course of ECHS1D, metabolic abnormalities at any step in the pathway may lead to changes in metabolic demands [21].

Mutations in the *HIBCH* gene have recently also been described as being the cause of Leigh-like syndrome. Abnormalities in metabolites indicate that HIBCH deficiency affects valine metabolism. The clinical symptoms of HIBCH deficiency were developmental delay, regression after an acute viral infection, dystonia, and bilateral basal ganglia abnormalities observed in MRI brain images. In the valine catabolic pathway, HIBCH is involved in the preceding step of ECHS1. The defect of HIBCH leads to metabolic pathway blockade and metabolite (substrate and methacrylyl-CoA) accumulation. Excess methacrylyl-CoA may react with thiol compounds, while cysteine residues, which are essential to mitochondrial enzymes, translate to thiol conjugates of methacrylyl-CoA. This series of changes caused a reduction in the cellular reduction state and ATP production, resulting in nerve cells in the basal ganglia [24]. ECHS1 and HIBCH deficiencies have many similarities in clinical and biochemical characteristics. Both can manifest feeding difficulties from the neonatal period, psychomotor retardation, dystonia, lactic acidosis, bilateral basal ganglia signal abnormalities on brain MRI, and sharp aggravation of the condition after acute viral infection [7].

The first time *ECHS1* mutations were identified as one of the causes of LS was in 2014, thanks to the rapid progress of the clinical application of next-generation sequencing technology. To date, more than thirty mutations from over 40 families have already been discovered. Here, we followed the same strategy as most previous works, implementing WES for the discovery of mutations and Sanger sequencing for mutation verification. Our findings enriched both the current mutation spectrum of the *ECHS1* gene and the phenotypic presentations of ECHS1D, especially in the Chinese population, as well as highlighting the importance of the valine catabolic pathway in Leigh syndrome.

## Data Availability

The datasets analysed during the current study are available in the NCBI Sequence Read Archive (SRA) repository [SRA accession: PRJNA637796, https://www.ncbi.nlm.nih.gov/sra/PRJNA637796]. The humanG1Kv37 (human_g1k_v37.fasta, MD5sum: 0ce84c872fc0072a885926823dcd0338) reference is equivalent to b37, with the exception that it does not contain the decoy sequence for human herpesvirus 4 type 1 (named NC007605_). This reference grew out of the 1000 Genomes Project. http://ftp.1000genomes.ebi.ac.uk/vol1/ftp/technical/reference/human_g1k_v37.fasta.gz.

## Abbreviations

ECHS1D: Short-chain enoyl-CoA hydratase deficiency
LS: Leigh syndrome
WES: Whole-exome sequencing
MRI: Magnetic resonance imaging
EEG: Electroencephalography
FAO: Fatty acid beta-oxidation
MRC: Mitochondrial respiratory chain
CS: Citrate synthase
OCR: Oxygen consumption rate
FCCP: Carbonyl cyanide phenylhydrazone
MRR: Maximum respiration rate
MAF: Minor allele frequency
